# Comparable biomechanical performance of magnesium-based and titanium screws for the Latarjet procedure in a cadaveric study

**DOI:** 10.1016/j.jseint.2025.101410

**Published:** 2025-12-11

**Authors:** Farah Selman, Esteban Ongini, Nicholas Peter James Perry, Michel Meisterhans, Maximilian Gressl, Karl Wieser

**Affiliations:** a Department of Orthopedics, Balgrist University Hospital, Zurich, Switzerland; b Institute for Biomechanics, ETH Zurich, Zurich, Switzerland; c Department of Orthopedic Surgery, Naval Medical Center San Diego, San Diego, CA, USA

**Keywords:** Magnesium screws, Biodegradable, Latarjet, Bone block fixation, Coracoid fixation, Biomechanical study

## Abstract

**Background and Hypothesis:**

Nonbiodegradable metal screws used for fixation in the Latarjet procedure can cause complications, including irritation from prominent hardware and the need for revision surgery. Magnesium-based, bioabsorbable screws may address these concerns. This study compared the biomechanical performance of magnesium screws to titanium screws with the hypothesis that there is no statistically significant difference between both groups.

**Methods:**

Fourteen fresh-frozen cadaveric shoulders were matched by age and sex. Seven shoulders underwent a standard Latarjet procedure using two 5 mm cannulated partially threaded magnesium screws (average age 70.6 ± 9.4 years). The other 7 were fixed with two 4.5 mm cannulated partially threaded titanium screws (average age 71.4 ± 14). All shoulders underwent biomechanical testing with direct pressure on the graft: cyclic loading (100 cycles 10 N- 100 N) was performed to assess displacement, stiffness and load-to-failure. Those were measured using force-displacement data obtained through a mechanical testing system.

**Results:**

Maximum cyclic displacement was 1.2 ± 0.6 mm for magnesium and 1.4 ± 1.1 mm for titanium screws (*P* = .643). Cyclical stiffness was 329 ± 147 N/mm (magnesium) vs. 402 ± 220 N/mm (titanium, *P* = .489). Construct stiffness was 327 ± 141 N/mm vs. 408 ± 207 N/mm (*P* = .425), and ultimate load to failure was 414 ± 128 N vs. 475 ± 271 N (*P* = .605). Bone block failure with screw bending was the most common failure mode in both groups.

**Conclusion:**

No statistically significant differences in biomechanical performance were found between magnesium and titanium screws in this time-zero biomechanical study. Magnesium screws may offer a viable bioabsorbable alternative that reduces complications from permanent hardware.

Anterior shoulder instability can be associated with glenoid bone loss, necessitating surgical intervention to restore stability.^4^ Bony reconstructive procedures like the Latarjet coracoid transfer^26^ are critically important tools to address glenohumeral instability. The aim is to enlarge the glenohumeral contact area and provide static stabilization.^4^^,^^10^^,^^13^^,^^34^^,^^35^ The Latarjet procedure, first described by Michel Latarjet in 1954, involves transferring the coracoid bone block to the anterior glenoid, achieving both static stabilization and dynamic stabilization through the conjoint tendon sling effect.^26^

Initially, Michel Latarjet described the use of one metal screw for bone block fixation. Over time, most surgeons adopted the use of 2 metal screws to achieve more rigid fixation and better union rates.^3^^,^^22^^,^^47^ Despite the procedure's good long-term outcomes, including low rates of recurrent instability and high patient satisfaction,^2^^,^^10^^,^^13^^,^^33^ complication rates as high as 30% have been reported.^18^^,^^19^^,^^32^^,^^37^^,^^38^ These complications include infection, screw loosening, migration and breakage, nerve irritation, and glenohumeral cartilage lesions, largely attributed to the metal properties of traditional screws.^37^^,^^50^ Furthermore, advanced imaging is often impaired by metal artifacts, complicating the evaluation of screw position and graft union. In some cases, symptomatic hardware or mispositioned screws necessitate reoperation, as highlighted by Griesser et al, who reported a reoperation rate of 7%, with 35% involving hardware removal.^18^

To address these challenges, bioabsorbable magnesium-based screws represent an innovative alternative. These screws avoid many of the drawbacks of traditional stainless steel or titanium screws, such as imaging artifacts, the need for hardware removal, and the creation of bony voids after removal. Magnesium-based screws are completely reabsorbed within one to two years, generate low imaging artifacts, and exhibit biomechanical properties comparable to cortical bone.^9^^,^^15^^,^^41^ While magnesium implants have been extensively used in maxillofacial surgery, they are gaining traction in orthopedics for applications such as fixation of tibial tubercle osteotomy, osteochondral fragment stabilization, and fixation of malleolar and radial head fractures.^1^^,^^12^^,^^27^^,^^42^^,^^43^ The exact strength and resorption rates of these implants vary depending on the alloy composition and proprietary coatings, necessitating biomechanical testing for new orthopedic applications.

Rigid fixation remains essential for achieving bony union in the Latarjet procedure. However, alternative techniques, such as non-rigid suture fixation, have been explored, particularly in arthroscopic procedures.^11^ These alternatives, while avoiding the drawbacks of metal screws, present their own limitations.^21^^,^^28^^,^^37^ Magnesium-based screws may offer a balanced solution by providing adequate initial biomechanical strength while eliminating the complications associated with metal hardware.

The purpose of this study is to compare the biomechanical performance of magnesium-based alloy screws to traditional titanium screws in a cadaveric Latarjet model. The hypothesis was that there would be no statistically significant difference in load-to-failure between the 2 screw types, supporting the viability of magnesium-based screws as a biomechanical safe and effective alternative for anterior shoulder stabilization.

## Materials and methods

A total of 14 fresh-frozen cadaveric shoulders were matched based on age and sex (Table I). Specimens with a history of shoulder surgery were excluded. Seven shoulders underwent the Latarjet procedure using two 5 mm cannulated partially threaded headless magnesium screws (group magnesium; average age 70.6 ± 9.4). The other 7 were fixed with two 4.5 mm cannulated partially threaded titanium screws (group titanium; average age 71.4 ± 14, *P* = .84).

**Table I:** Baseline demographics matched specimens.

Nr	Group	Sex	Age [yr]	
1	Mg+	M	73	
2	Mg+	F	51	
3	Mg+	M	71	
4	Mg+	F	69	
5	Mg+	M	77	
6	Mg+	F	73	
7	Mg+	M	80	
		**Mean**	**70.57**	
		SD	9.38	
				*P* = .896
8	Titan	F	51	
9	Titan	F	61	
10	Titan	M	70	
11	Titan	F	70	
12	Titan	M	76	
13	Titan	M	97	
14	Titan	M	75	
		**Mean**	**71.43**	
		SD	14.25	

*SD*, standard deviation; *Mg*, magnesium; *Titan*, titanium.

### Specimen preparation

All specimens were prepared by a single orthopedic surgery resident (F.S.) with the help of a medical student (M.G.) under the supervision of a specialized shoulder and elbow surgeon (K.W.).

Preparation was the same between groups until the drilling for screw insertion:

The scapula was freed from all surrounding soft tissues, including the conjoint tendon. The osseous scapular body was shaped with an oscillating saw (Smith & Nephew, Andover, MA, USA) in order to fit into a custom-designed rectangular box. The specimens were securely embedded using polymethyl methacrylate for stabilization.

The anterio-inferior labrum was sharply excised from the glenoid. A glenoid osteotomy was performed in the corresponding quadrant, mimicking a defect of 25% anterior bone loss.^39^^,^^47^

A coracoid osteotomy was performed with an oscillating saw at 25 mm from the tip of the coracoid. The concave surface of the coracoid bone block was flattened with a saw to create a flush surface to match the glenoid. With a specifically designed small Meyer Latarjet Drill Guide (Innomed, Cham, Switzerland) two 3.5 mm parallel holes were drilled 10 mm apart according to the manufacturer's recommendations for both groups. The graft was placed flush with the anterio-inferior glenoid rim. With two 3 mm K-Wires holding the coracoid graft temporarily in place, 2 corresponding holes were drilled in the glenoid, using the Meyer Latarjet Drill Guide.

For the magnesium group two 5 mm, cannulated, partially threaded, headless magnesium screws of 36 mm length (Medical Magnesium, Achen, Germany) were used to fix the coracoid bloc. The other 7 coracoids were fixed with each two 4.5 mm, cannulated, partially threaded titanium screws of 36 mm length (Arthrex, Naples, FL, USA).

### Biomechanical testing

**Figure 1:**
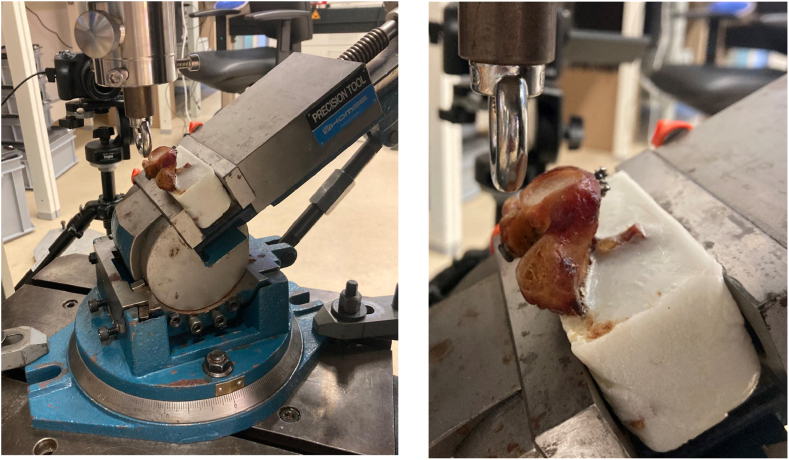
Testing protocol with direct load of the graft.

The testing protocol included a direct load on the graft, adapted from previously published studies^3^^,^^5^^,^^39^^,^^47^ (Fig. 1). The glenoid was fixed in 30° anteversion on the testing machine (Zwick 1456; Zwick/Roell, Ulm, Germany). Load was applied by a metallic ring with a diameter of 36 mm and a width of 8 mm attached to a 20 kN load cell (20 kN Serie K; GTM Testing and Metrology GmbH, Birkenbach, Germany). The direct pressure on the middle of the graft coupled with the 30° of anteversion intended to simulate a worst-case clinical scenario as described before.^3^^,^^23^^,^^39^^,^^47^

**Figure 2:**
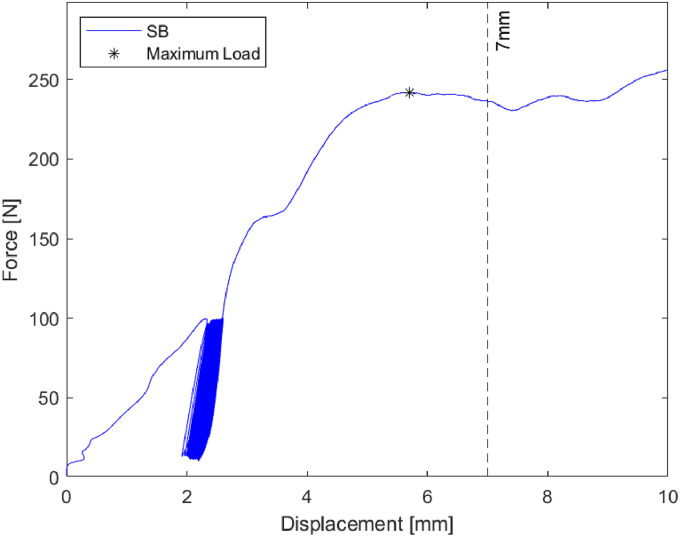
Force-displacement curve of the bone block after 100 cycles, followed by monotonically increasing load until failure (defined as ≥7 mm displacement).

After preloading each specimen to 1 N over a period of 5 seconds to ensure consistent contact with the graft, all grafts were cyclically loaded from 10 N to 100 N at a rate of 1 Hz for 100 cycles. After 100 cycles, each bone block was monotonically loaded until failure, which was defined as ≥7 mm displacement (Fig. 2). Displacement was measured directly by the testing machine using the crosshead displacement data, which reflects the relative movement between the loading ring and the fixed glenoid block.

In addition to biomechanical testing, the mode of failure was assessed for each specimen. After load-to-failure testing, the screws were carefully removed and both the screws and bone block were visually examined and photo-documented to characterize the failure pattern (eg, screw breakage, graft fracture, screw loosening, etc.).

### Outcome measures

The maximum displacement observed during cycling loading was documented. For cyclic loading tests, the maximum cycle stiffness of the constructs was determined by calculating the slope of the loading curve at the 100th cycle. In failure testing, the peak load necessary to induce failure was measured. The stiffness of the constructs was derived by calculating the slope within the linear portion of the curve. All mechanical parameters were computed using MATLAB (version 2021a; MathWorks, Inc., Natick, MA, USA).

### Statistical analysis

The Shapiro-Wilk test was used to assess the normality of all outcome measures. Continuous variables were reported as mean and standard deviation. Differences between fixation groups were analyzed using a 2-tailed unpaired *t*-test, with statistical significance set a *P* < .05. Data analysis was conducted using GraphPad Prism (version 9.2.0 for Windows; GraphPad Software LLC, San Diego, CA, USA).

## Results

**Figure 3:**
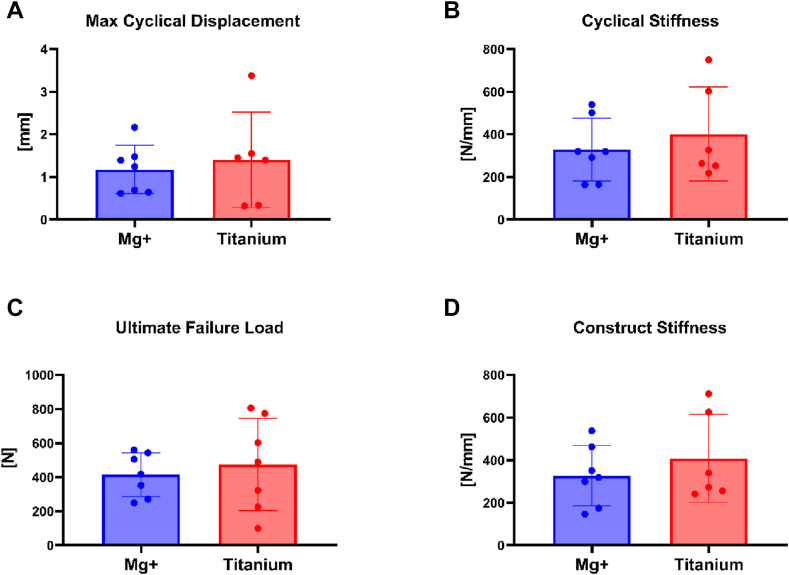
No significant differences were observed between the magnesium and titanium groups across all biomechanical tests.

There were no significant differences in all tests between the magnesium and titanium group (Fig. 3).

### Displacement during cyclic loading

During cyclic loading from 10N to 100N, the maximum displacement was not significantly different for the magnesium group compared to the titanium group (magnesium 1.18 ± 0.57 mm, 95% confidence interval [CI] 0.65-1.70; titanium 1.40 ± 1.12 mm, 95% CI 0.23-2.58; *P* = .643).

### Stiffness during 100th cycle

During the 100th cycle, before initiating the load to failure, there was no significant difference in the stiffness of the magnesium group compared to the titanium group. (magnesium 329 ± 147 N/mm, 95% CI 193-465; titanium 402 ± 220 N/mm, 95% CI 172-633; *P* = .489).

### Ultimate load to failure

During load to failure, the ultimate load was not significantly different for the magnesium group compared to the titanium group (group magnesium mean 414 ± 128 N, 95% CI 296-532; group titanium mean 475 ± 271 N, 95% CI 224-725; *P* = .605). Failure was defined as maximum graft displacement of 7 mm.

### Stiffness during load to failure

During load to failure, the overall stiffness of the construct was not significantly different for the magnesium group compared to the titanium group (magnesium 327 ± 141 N/mm, 95% CI 197-458; titanium 408 ± 207 N/mm, 95% CI 191-625; *P* = .425).

### Failure mode

**Figure 4:**
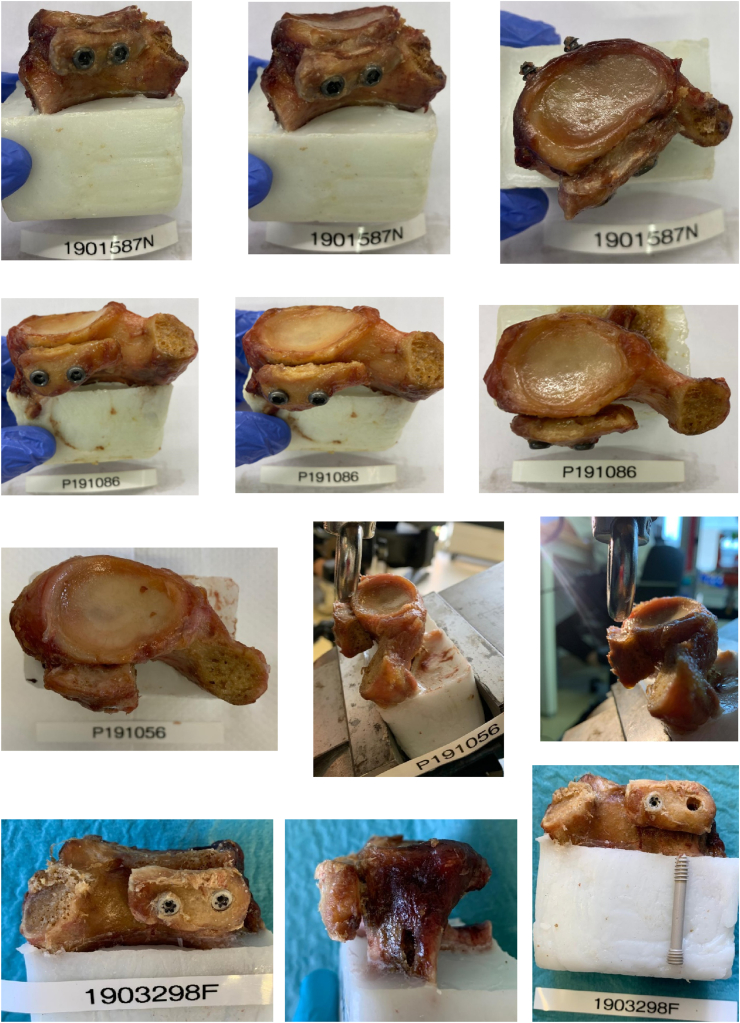
Bone block compression deformity was the most common failure mode in both groups. The *upper* 6 images show titanium screw fixation, while the *lower* 6 images depict magnesium screw fixation.

**Figure 5:**
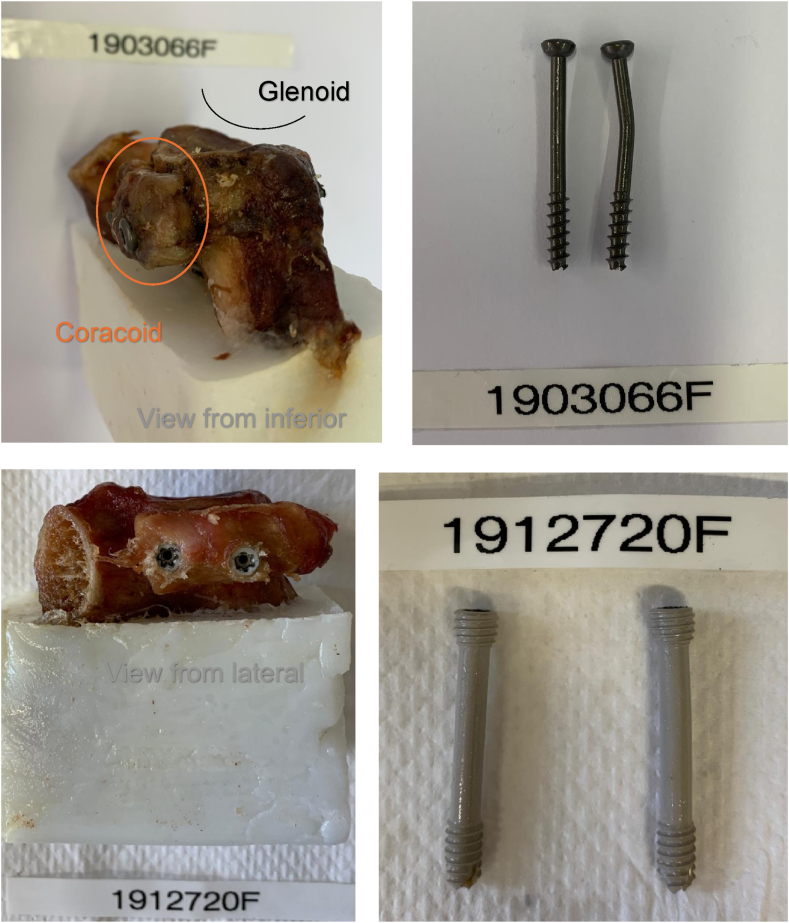
Bone block compression deformity was the most common failure mode in both groups, with screw bending observed in some cases. The *upper* 2 images show bent titanium screws, while the *lower* 2 images show bent magnesium screws.

The most common failure mode in both groups was bone block compression deformity (Fig. 4) with screw bending in some cases (Fig. 5).

## Discussion

The most important finding of this biomechanical study is that the tested magnesium-based screws showed similar performance for the ultimate load to failure and cyclic displacement of the coracoid graft compared to the titanium screw fixation.

The Latarjet coracoid transfer procedure is a reliable method to restore glenohumeral stability but the use of nonabsorbable, rigid screw fixation, can lead to secondary issues. Screw heads often become prominent as the coracoid bone block resorbs postoperatively, losing approximately 42% of its volume and 2.2 mm in width over 24 months.^36^^,^^44^ This mismatch between bone block resorption and the nonabsorption of metal screws may contribute to hardware-related complications and revision rates up to 3.7%-7%.^18^^,^^24^ Gilat et al^16^ reported similar results, that the majority of complications (1.6%) were related to hardware failure or removal using standard metal screw fixation. These complications include screw pullout, breakage, migration, bone block fracture, nonunion, and graft resorption. It has been demonstrated that screw removal alleviates pain in patients with persistent shoulder pain after the Latarjet procedure. Godenèche et al^17^ analyzed 461 shoulders treated with the Latarjet procedure and found that 21 cases (4.6%) experienced anterior focal pain without instability recurrence. Among these, screw removal successfully resolved pain in 67% of shoulders (N = 14) and led to pain reduction in the remaining 7 cases (33%).

Nondegradable metal screws in the Latarjet procedure can lead to graft osteolysis.^20^^,^^49^ Even if initially well-placed, the screw heads may become prominent due to postoperative bone resorption, leading to irritation of the surrounding soft tissues. This condition can potentially cause complications such as subscapularis tearing, humeral head impingement, glenohumeral osteoarthritis, and the mentioned pain.^31^ In this context, the use of stable headless screws without overhang may reduce irritation and pain of the surrounding soft tissue, leading to a lower revision rate.

Whether screws are removed for a revision glenoid reconstruction procedure or conversion to a shoulder arthroplasty, the resulting bony void may contribute to difficult or poor fixation of implants in the secondary procedure. Finally, the screws consistently cause significant artifacts on advanced imaging making it difficult to assess implant positioning, bone block positioning at the joint, or for the presence of progressing cartilage loss and eventual osteoarthritis.

To avoid some of the issues of nonabsorbable, traditional metal screws and to facilitate arthroscopic techniques, some authors have advocated for suture button fixation.^11^ Suture button fixation has the advantage of minimizing the amount of permanent metal in the shoulder and avoiding any residual metal in the glenoid vault. However, the combination of losing rigid fixation and graft absorption could potentially lead to compromising clinical outcomes.^22^^,^^28^

Magnesium-based alloy screws and other implants potentially could offer a unique solution to some of these issues. These screws provide time-zero rigid fixation with a modulus of elasticity similar to cortical bone, which may prevent stress shielding. Without the heavy metals and magnetic properties of traditional metal screws, magnesium-based screws produce minimal artifacts on advanced imaging.^40^^,^^41^ They are typically reabsorbed within a 1-2 year period and are replaced with host-generated bone. Various factors such as exact magnesium alloy composition, proprietary surface coating, and local in vivo biochemical and biomechanical affects the exact rate of reabsorption.^9^^,^^15^ It is crucial for the orthopedic community to recognize that not all "magnesium screws" are equivalent. As new alloys are explored, it is essential to avoid treating them as a single, homogeneous group.

Basic science and animal studies suggest other potential advantages for magnesium-based implants. The byproducts of screw reabsorption may have immunomodulatory and proangiogenic properties, which promote bone healing and decrease infection.^8^^,^^46^ In vivo animal studies have histologically demonstrated that the newly formed bone is similar to native bone, supporting the claim of osteoconductive properties.^14^^,^^29^ Finally, animal models have also suggested that magnesium-alloy based implants may actually improve graft incorporation in a soft tissue ACL reconstruction model.^45^

Magnesium-based alloy implants have several current clinical applications. A recent retrospective review of medial malleolus ankle fracture fixation using magnesium-based screws compared to titanium screws demonstrated equal rates of bony union and a decreased rate of hardware removal for the magnesium group.^30^ Another study of 10 patients undergoing a tibial tubercle osteotomy with fixation using 4.8 mm magnesium-based screws demonstrated excellent imaging characteristics on CT and gradual reabsorption of the screws in humans without compromising the osteotomy site.^43^ Baldini et al demonstrated the safe and effective use of magnesium screws for fixation of medial epicondyle fractures across an active physis while avoiding the need for hardware removal.^6^^,^^7^ A prospective, randomized study of magnesium versus titanium in fixation of chevron osteotomy for surgical treatment of hallux valgus showed no difference between the 2 groups, though cohorts were small and follow-up was short.^48^ Finally, an ongoing prospective, randomized trial is comparing magnesium-based screws to titanium screws for treatment of scaphoid fractures.^25^ Magnesium-based alloy implants are not currently FDA approved, and their use is limited in the US, though several companies are focused on working through these regulatory hurdles.

### Limitations

This study utilizes a cadaveric model with high ages of the cadavers at the time of death (ranging from 74 to 96 years), which differs significantly from the typically younger population treated for anterior shoulder instability. This discrepancy in bone quality may impact the study's results. Additionally, the forces applied to the transferred coracoid during rehabilitation remain unknown, making it unclear whether the stronger fixation offered by bicortical metal screws is truly necessary.

Another limitation of this study is that the screw diameters were not identical between the magnesium and titanium screws. Since screw strength is related to radius, even small differences in diameter may have influenced the biomechanical results.

A complete release of the subscapularis and conjoint tendons was performed to facilitate the procedure and enhance reproducibility; however, this adjustment should not influence the evaluation of the coracoid fixation strength.

## Conclusion

The findings of this cadaveric study demonstrated no statistically significant differences in biomechanical parameters, including cyclic displacement, construct stiffness, and ultimate load to failure, between magnesium-based and titanium screw fixation in glenoid bone block constructs. Magnesium screws may offer a bioabsorbable alternative to titanium screws, potentially reducing hardware-related complications.

## Disclaimers:

Funding: This work was supported by a contribution from the Swiss Orthopaedics Research Fund.

Conflicts of interest: The authors, their immediate families, and any research foundation with which they are affiliated have not received any financial payments or other benefits from any commercial entity related to the subject of this article.
